# Supplementary material for: Distribution of Biodiversity of Wild Beet Species (Genus Beta L.) in Armenia under Ongoing Climate Change Conditions
Source: Plants (Basel). 2022 Sep 24;11(19):2502. doi: 10.3390/plants11192502 (PMC9573691; doi:10.3390/plants11192502)
Supplement: Supplementary file 1 [file plants-11-02502-s001.zip › Figure S1.pdf]

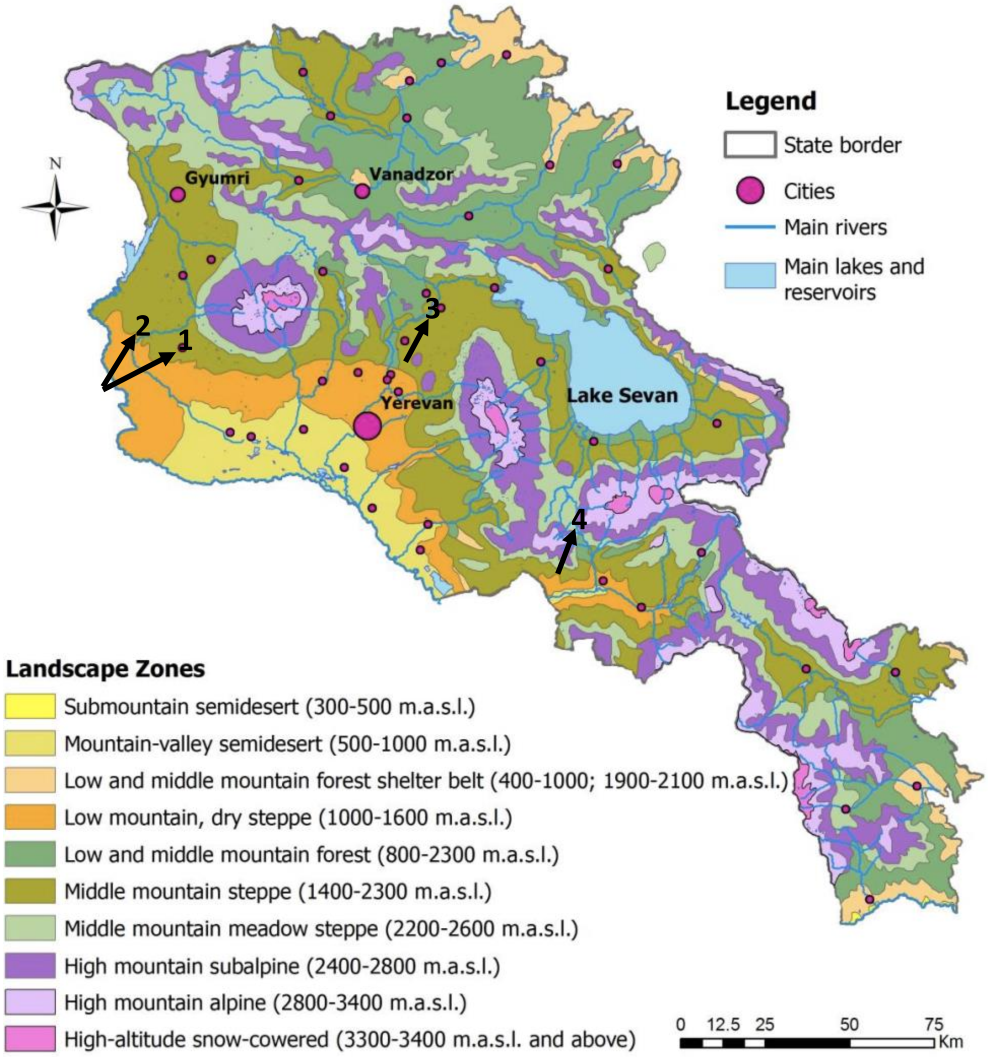

Plot	Location	Altitude m a.s.l	Landscape zone	Species
1	Akunq	1700	Middle mountain steppe	*B. lomatogona*
2	Zarindja	1660		
3	Hrazdan	1665	Middle mountain steppe	*B. corolliflora*
4	Vardenyats Pass	2410	Middle mountain meadow steppe	*B. macrorhiza*

Figure S1. Landscape zones of the Republic of Armenia. Selected locations of *B. lomatogona*, *B. corolliflora* and *B. macrorhiza*. Adapted from Fifth National Report of the Republic of Armenia to the Convention on Biological Diversity; Yerevan, Armenia, 2014; 107 p. URL: https://www.cbd.int/doc/world/am/am-nr-05-en.pdf
