# Supplementary material for: Distribution of Biodiversity of Wild Beet Species (Genus Beta L.) in Armenia under Ongoing Climate Change Conditions
Source: Plants (Basel). 2022 Sep 24;11(19):2502. doi: 10.3390/plants11192502 (PMC9573691; doi:10.3390/plants11192502)
Supplement: Supplementary file 1 [file plants-11-02502-s001.zip › Figure S2.pdf]

A

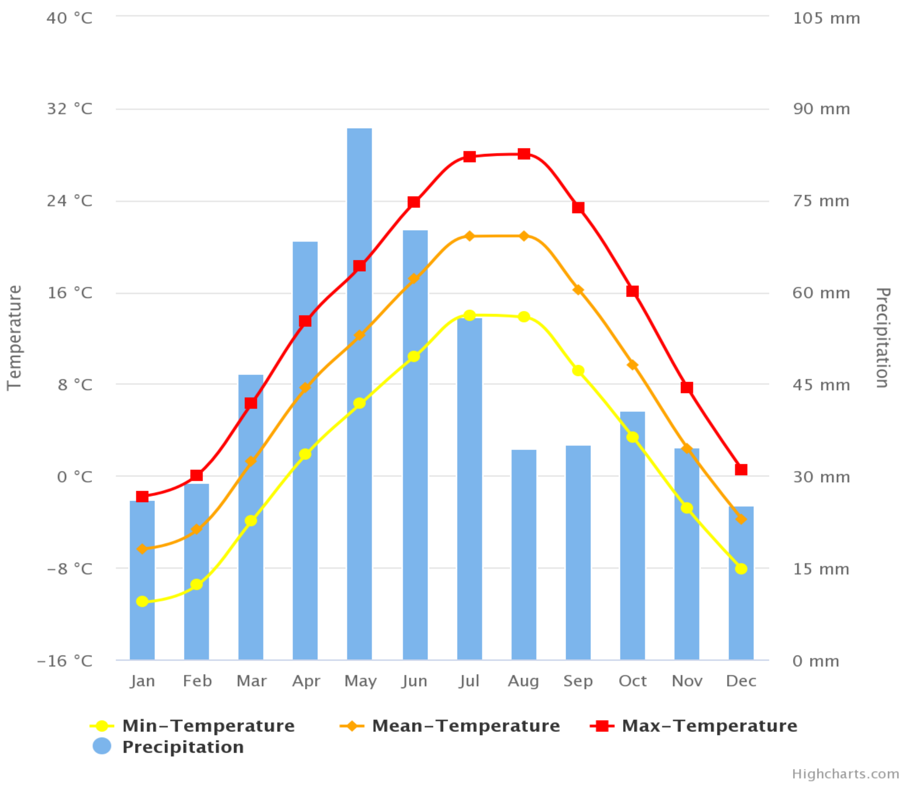

B

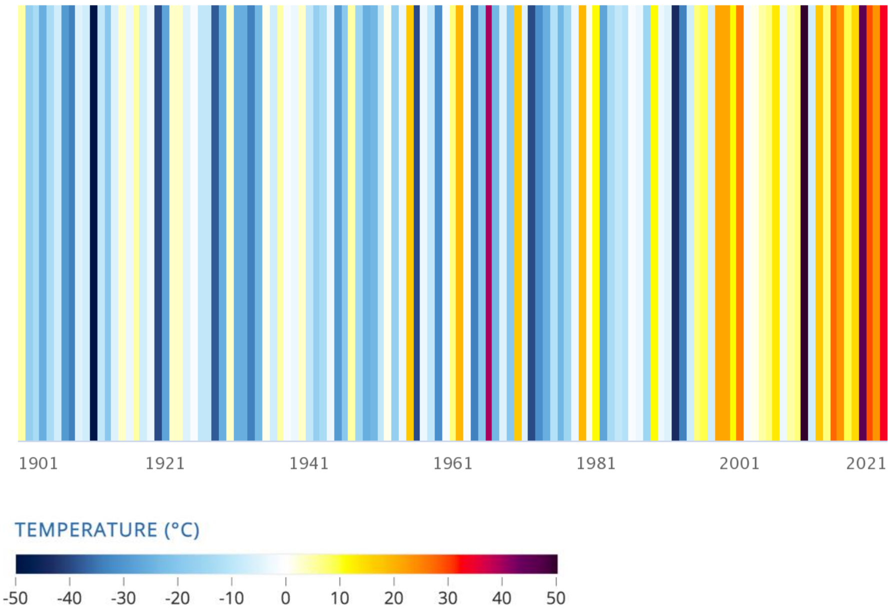

Figure S2. Climate in Armenia. Monthly climatology of min-temperature, mean temperature, max-temperature and precipitation in 1991-2020 in Armenia **(A).** Observed annual mean-temperatures, 1901-2020 in Armenia **(B).** Obtained from WBG Climate Change Knowledge Portal (CCKP, 2020). Climate Data: Historical. URL: https://climateknowledgeportal.worldbank.org/country/armenia/climate-data-historical
