# Supplementary material for: Distribution of Biodiversity of Wild Beet Species (Genus Beta L.) in Armenia under Ongoing Climate Change Conditions
Source: Plants (Basel). 2022 Sep 24;11(19):2502. doi: 10.3390/plants11192502 (PMC9573691; doi:10.3390/plants11192502)
Supplement: Supplementary file 1 [file plants-11-02502-s001.zip › Figure S3.pdf]

A. Distribution of *Beta lomatogona* Fisch. & C.A.Mey.

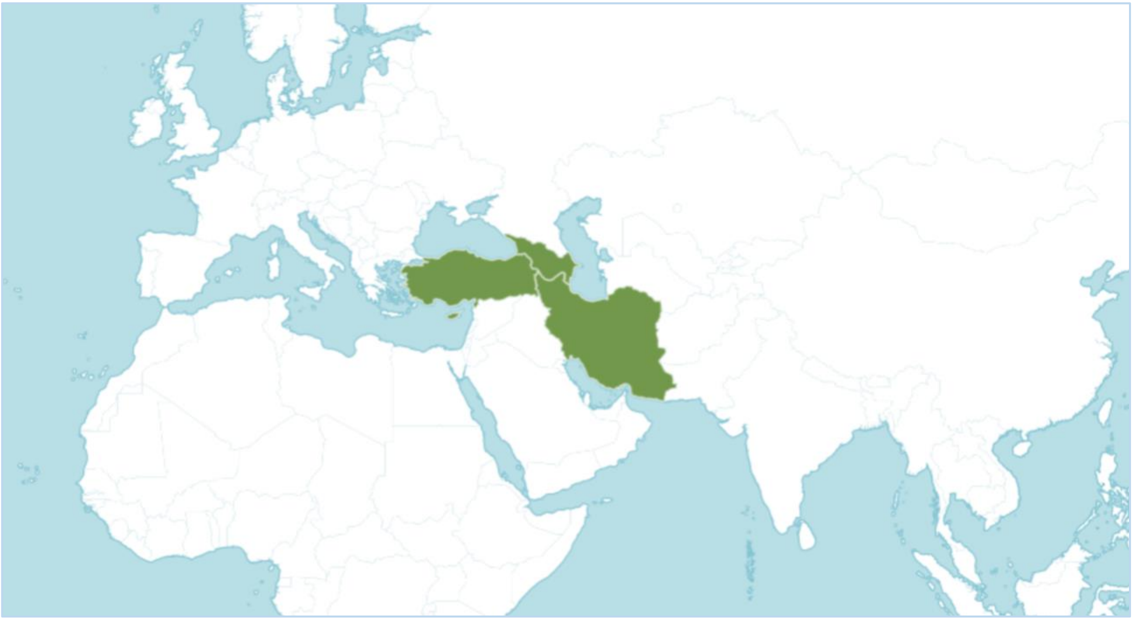

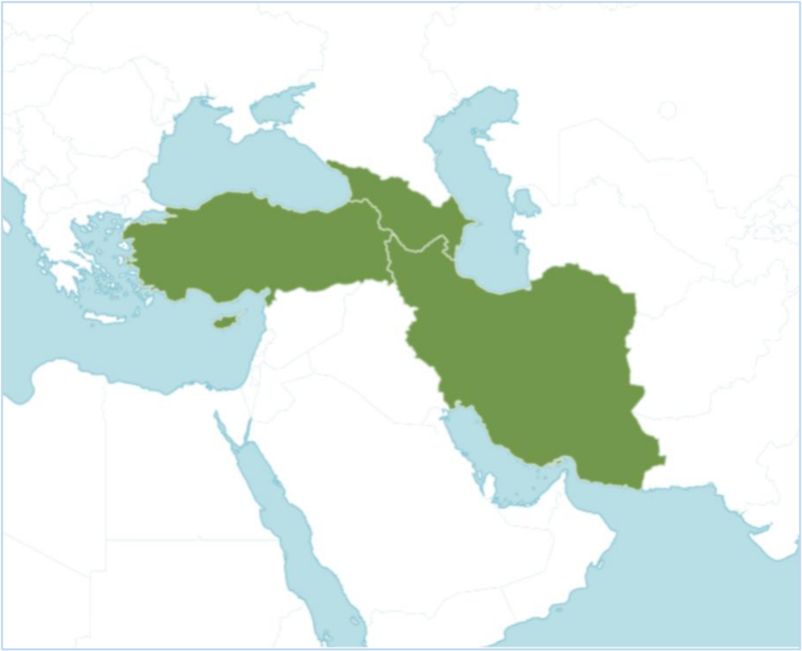

■ Native

Native to:

Cyprus, Iran, Transcaucasus, Turkey

Reference:

POWO (2022). "Plants of the World Online. Facilitated by the Royal Botanic Gardens, Kew. Published on the Internet; http://www.plantsoftheworldonline.org/
Retrieved 25 August 2022."

B. Distribution of *Beta corolliflora* Zosimovic ex Buttler

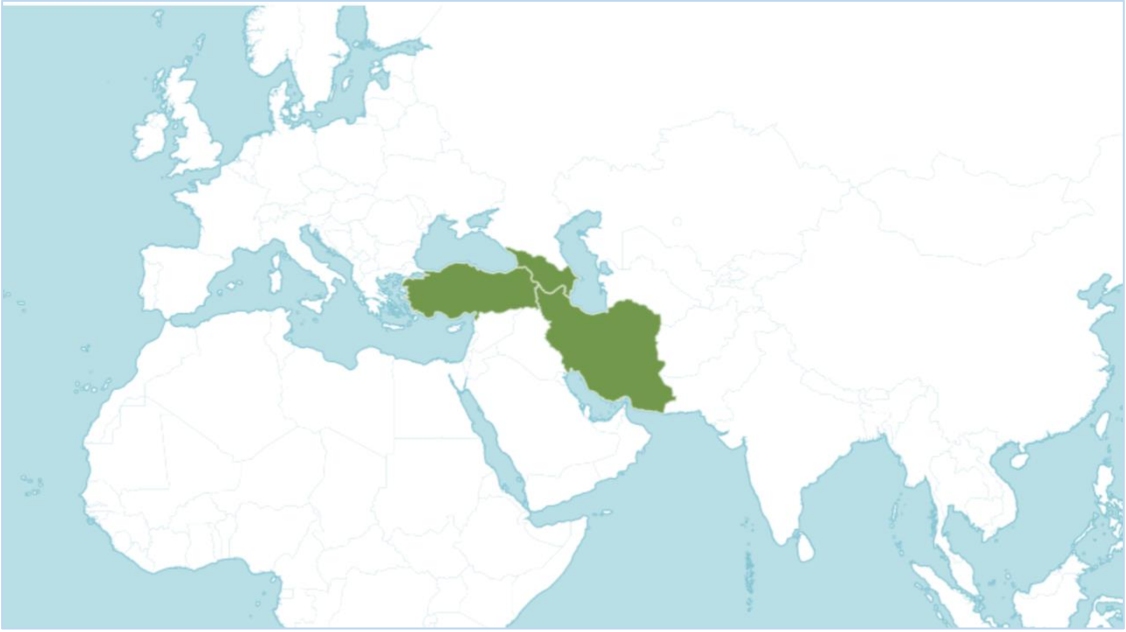

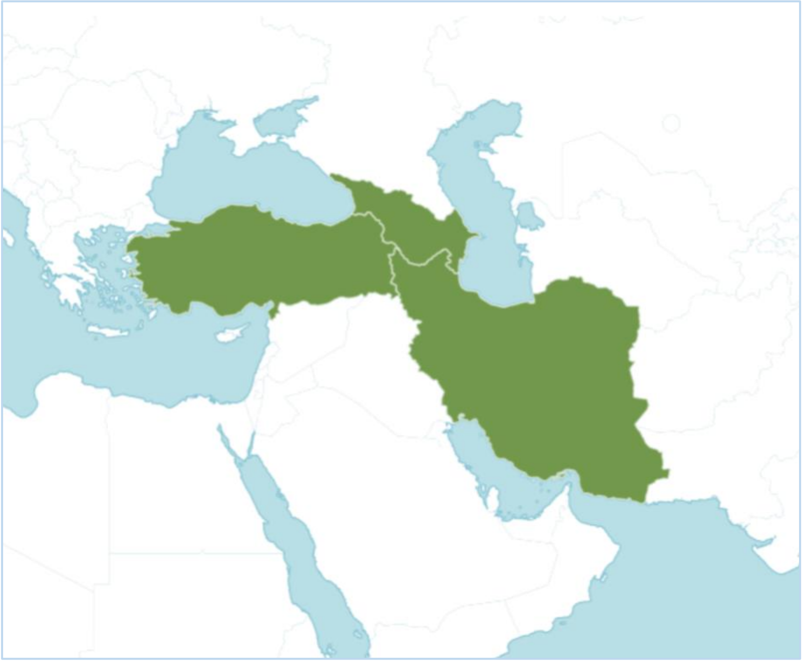

Native

Native to:

Iran, Transcaucasus, Turkey

Reference:

POWO (2022). "Plants of the World Online. Facilitated by the Royal Botanic Gardens, Kew. Published on the Internet; http://www.plantsoftheworldonline.org/
Retrieved 25 August 2022.

C. Distribution of *Beta macrorhiza* Steven

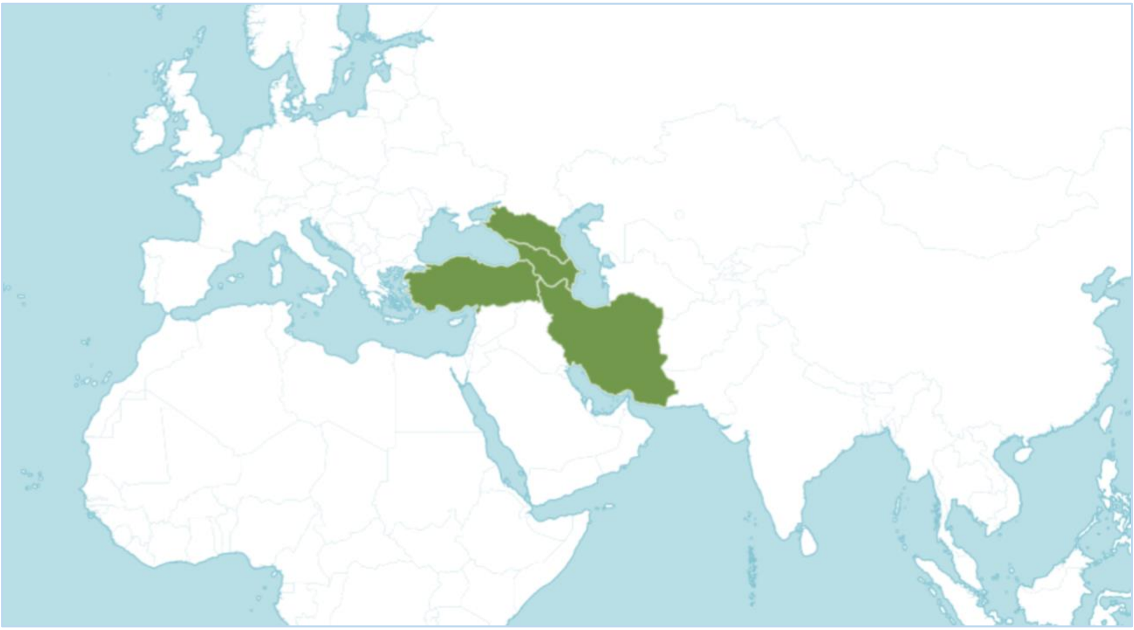

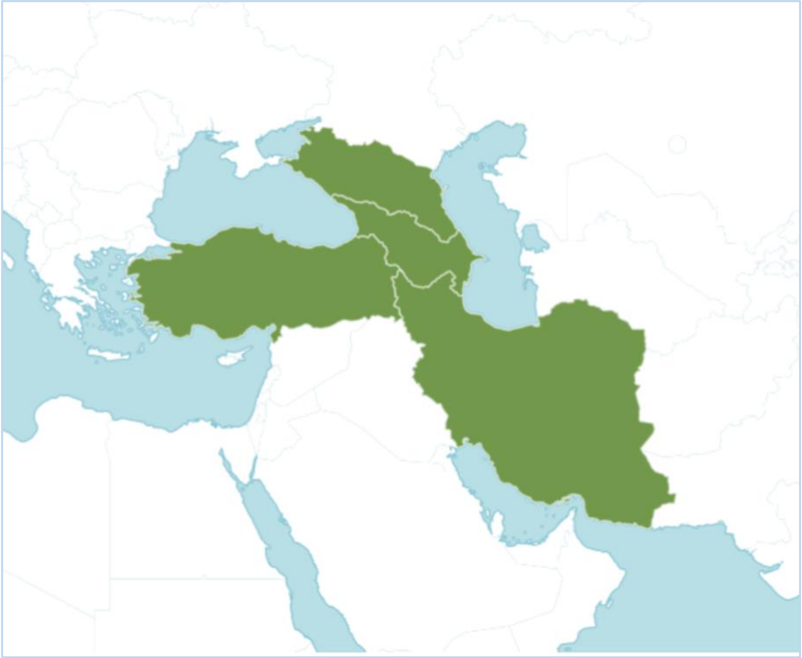

■ Native

Native to:

Iran, North Caucasus, Transcaucasus, Turkey

Reference:

POWO (2022). "Plants of the World Online. Facilitated by the Royal Botanic Gardens, Kew. Published on the Internet; http://www.plantsoftheworldonline.org/
Retrieved 25 August 2022.

Figure S3: Distribution range of *B. lomatogona*, *B. corolliflora* and *B. macrorhiza*.
