# Supplementary material for: Distribution of Biodiversity of Wild Beet Species (Genus Beta L.) in Armenia under Ongoing Climate Change Conditions
Source: Plants (Basel). 2022 Sep 24;11(19):2502. doi: 10.3390/plants11192502 (PMC9573691; doi:10.3390/plants11192502)
Supplement: Supplementary file 1 [file Table S1.pdf › Table S1.pdf]

Table S1. Total precipitation and average temperature during winter (A) and summer (B) periods in the selected regions of Aragatsotn, Gegharkunik and Kotayk marzes in 2010-2020.

	Total precipitation December-March (mm)			Average temperature December-March (°C)		
**Years**	**Aragatsotn**	**Kotayk**	**Gegharkunik**	**Aragatsotn**	**Kotayk**	**Gegharkunik**
2010-2011	123.9	**245.1**	**142.5**	2.1	-3.5	-3.9
2011-2012	103.1	207.0	82.8	-1.2	-6.6	-6.8
2012-2013	147.3	**356.9**	105.6	2.4	-3.4	-2.9
2013-2014	95.0	219.0	**123.2**	-0.7	-5.1	-5.1
2014-2015	84.7	172.2	**124.2**	3.0	-2.2	-2.8
2015-2016	125.8	**280.0**	**141.2**	1.8	-3.2	-3.7
2016-2017	122.5	166.4	116.8	-3.9	-7.0	-6.7
2017-2018	106.2	**231.5**	93.2	**4.9**	-0.2	-0.5
2018-2019	**159.9**	**245.5**	**130.7**	**2.9**	-2.4	-3.4
2019-2020	**142.4**	197.2	100.5	**3.4**	-1.7	-2.2

(**A**)

	Total precipitation July-September (mm)			Average temperature July-September (°C)		
**Years**	**Aragatsotn**	**Kotayk**	**Gegharkunik**	**Aragatsotn**	**Kotayk**	**Gegharkunik**
2010	36.4	102.3	124.7	**25.5**	**18.1**	16.1
2011	53.0	101.9	145.4	23.9	16.8	14.9
2012	47.8	101.4	165.9	24.1	17.2	15.1
2013	**24.2**	**90.5**	**74.0**	23.0	15.7	14.3
2014	**29.2**	100.6	125.6	25.2	**17.7**	15.8
2015	**27.4**	**64.9**	**90.5**	**25.9**	**18.2**	16.1
2016	72.8	148.5	133.9	23.8	16.2	14.7
2017	**31.1**	**60.2**	**86.6**	**26.6**	**18.7**	**16.8**
2018	**20.2**	**47.7**	150.7	**25.5**	**17.9**	16.1
2019	38.4	**80.7**	145.5	24.3	16.5	14.9
2020	37.7	**80.7**	146.0	24.5	15.9	16.1

(**B**)
